# A Novel Micromachined Z-axis Torsional Accelerometer Based on the Tunneling Magnetoresistive Effect

**DOI:** 10.3390/mi11040422

**Published:** 2020-04-17

**Authors:** Bo Yang, Xiaoyong Gao, Cheng Li

**Affiliations:** 1 School of Instrument Science and Engineering, Southeast University, Nanjing 210096, China; 220173252@seu.edu.cn (X.G.); 230198302@seu.edu.cn (C.L.); 2 Key Laboratory of Micro-Inertial Instrument and Advanced Navigation Technology, Ministry of Education, Nanjing 210096, China

**Keywords:** accelerometer, tunneling magnetoresistive effect, torsional structure, electrostatic force feedback

## Abstract

A novel micromachined z-axis torsional accelerometer based on the tunneling magnetoresistive effect is presented in this paper. The plane main structure bonded with permanent magnetic film is driven to twist under the action of inertial acceleration, which results in the opposite variation of the magnetic field intensity. The variation of the magnetic field is measured by two differential tunneling magnetoresistive sensors arranged on the top substrate respectively. Electrostatic feedback electrodes plated on the bottom substrate are used to revert the plane main structure to an equilibrium state and realize the closed-loop detection of acceleration. A modal simulation of the micromachined z-axis tunneling magnetoresistive accelerometer was implemented to verify the theoretical formula and the structural optimization. Simultaneously, the characteristics of the magnetic field were analyzed to optimize the layout of the tunneling magnetoresistance accelerometer by finite element simulation. The plane main structure, fabricated with the process of standard deep dry silicon on glass (DDSOG), had dimensions of 8000 μm (length) × 8000 μm (width) × 120μm (height). A prototype of the micromachined z-axis tunneling magnetoresistive accelerometer was produced by micro-assembly of the plane main structure with the tunneling magnetoresistive sensors. The experiment results demonstrate that the prototype has a maximal sensitivity of 1.7 mV/g and an acceleration resolution of 128 μg/Hz^0.5^ along the z-axis sensitive direction.

## 1. Introduction

Microelectromechanical (MEMS) inertial sensors are small, robust and cheap, hence ideal candidates for applications from navigation to automotive [1,2,3,4,5]. An accelerometer is a micromechanical sensor which measures various modes of accelerations whether they are constant (gravity), time-varying (vibrations), or quasi-static (tilt) [6,7,8,9,10]. To date, a variety of accelerometer solutions and sensitive principles have emerged and have been successfully commercialized for different fields [11,12,13,14,15,16]. However, many factors, including small proof mass, weak signals, low detection sensitivity and high noise interference, limit further improvement of MEMS accelerometers in performance [17,18].

Research on an accelerometer based on the tunnel magnetoresistive effect, which has been gradually favored by consumer applications owing to its ultra-high sensitivity, wide temperature operating range and robustness against various types of contamination, has received extensive attention due to the rapid development of magnetoresistive technology [19,20,21,22,23]. Olivas, J.-D, et. Al, in the National Aeronautics and Space Administration (NASA), proposed an ultrasensitive displacement-sensing device based on the magnetoresistive (GMR) effect in 2003, mainly used for signal detection of acceleration, pressure and temperature [24]. The structure consists of multilayer films based on the magnetoresistive film layer and the hard magnetic film layer. When the space between the magnetoresistive film layer and the hard magnetic film layer changes due to the acceleration, pressure or temperature, the resistance of the magnetoresistive film layer changes, thus realizing the signal detection. The scheme only gives the model in principle, without an actual prototype. A biaxial accelerometer based on the magnetoresistive (MR) effect, which has a proof mass fabricated by a mushroom-shaped polymer magnet, was presented at Eindhoven University of Technology in 2008 [25]. A minute movement under lateral acceleration is precisely sensed by a set of symmetrically arranged magnetoresistive (MR) sensors. The prototype achieved a sensitivity of 0.32 mV/g and a noise density of 35 μg/Hz^0.5^. A small tunneling magnetoresistive accelerometer based on 3D printing has been implemented with a bias stability of 64.40 μg in the literature [26]. However, due to the tolerance limitation of 3D processing technology, the volume of the entire device is too large, and it is difficult to further reduce the minimum gap, which limits the further improvement of sensitivity. A micromachined z-axis tunneling magnetoresistive accelerometer based on microfabrication and micro-assembly technology has been proposed, with a noise floor of 86.2 μg/Hz^0.5^, in the literature [27]. Another novel method of acceleration measurement was based on tunneling magnetoresistance, including a tunneling magnetoresistive (TMR) sensor that was used to precisely measure the magnetic field and a micro-cantilever beam bonded with the cylinder permanent magnet, which converted input acceleration to magnetic field changes. The accelerometer demonstrated a linear system response with a sensitivity of 1.145 V/g and was presented in the literature [28].

This paper presents a novel micromachined z-axis torsional accelerometer based on the tunneling magnetoresistive effect, which is mainly composed of the top substrate, the middle plane main structure and the bottom substrate. The middle plane main structure bonded with permanent magnetic film is driven to twist under the action of the inertial acceleration, which results in the opposite variation of the magnetic field intensity around two diagonal boundaries of the permanent magnetic film. The magnetic field variation is measured by two differential tunneling magnetoresistive sensors, which are arranged on the top substrate directly above two diagonal boundaries of the permanent magnetic film. Section 2 presents the structure principle of the micromachined z-axis tunneling magnetoresistive accelerometer. Then the simulation analysis and the measurement and control circuit are described in Section 3 and Section 4. We illustrate the experimental results in Section 5. Conclusions are finally given in the last section.

## 2. Structure Principle

**Figure 1:**
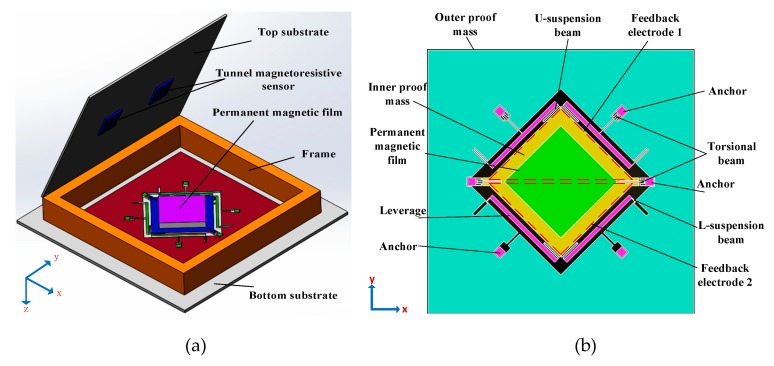
Structural schematic of the micromachined z-axis tunnel magnetoresistive accelerometer. (**a**) The micro-assembly structure layout of tunnel magnetoresistive accelerometer. (**b**) The plane main structure of tunnel magnetoresistive accelerometer.

The structural schematic of the micromachined z-axis tunneling magnetoresistive accelerometer is shown in Figure 1. The micromachined z-axis tunneling magnetoresistive accelerometer is composed of the top substrate, the plane main structure and the bottom substrate, as illustrated in Figure 1a. Two tunneling magnetoresistive sensors in opposite sensitive directions are arranged on the top substrate, directly above the two diagonal boundaries of the permanent magnetic film. The tunneling magnetoresistive sensors are used to detect the variation of the surrounding magnetic field caused by the input acceleration. The top substrate is bonded with the plane main structure in the frame using the micro-assembly method. The plane main structure is the principal component of the micromachined z-axis tunnel magnetoresistive accelerometer, and the specific layout of the structure is shown in Figure 1b. The plane main structure consists of two leverage structures, two linkage structures, a permanent magnetic film, an inner proof mass and an outer proof mass. The outer proof mass is connected to the inner proof mass through two leverage structures and two linkage structures. Simultaneously, the inner proof mass bonded with the permanent magnetic film is fixed at anchors by two torsion beams. Furthermore, two feedback electrodes with the same dimension are plated on the bottom substrate. When the acceleration is inputted along the z-axis, the outer proof mass moves straight along z-axis under the inertia force, which drives the inner proof mass to twist through leverage structures and linkage structures. The minute torsion of the permanent magnetic film induced by the inner proof mass results in the opposite variation of the magnetic field intensity around two diagonal boundaries of the permanent magnetic film. Two differential tunneling magnetoresistive sensors, which are arranged on the top substrate directly above two diagonal boundaries of the permanent magnetic film, are utilized to measure the magnetic field variation. Finally, the closed-loop control signal is imported to feedback electrodes to realize the closed-loop detection of the acceleration owing to the electrostatic force between the inner proof mass and feedback electrodes.

Compared with conventional commercial capacitive accelerometers, the tunneling magnetoresistive accelerometer uses the tunnel magnetoresistance effect to measure the input acceleration by sensing the variation of the magnetic field. The tunnel magnetoresistance effect, which utilizes the quantum tunneling effect to change the resistance, has higher displacement detection sensitivity compared with the capacitive detection method. At the same time, the traditional capacitive accelerometer is very sensitive to the surrounding parasitic capacitance due to the capacitive detective method. The tunneling magnetoresistive accelerometer is basically unaffected by the surrounding parasitic capacitance. Therefore, the tunneling magnetoresistive accelerometer has great potential and is expected to further improve the performance. The previous structure produced by our team, shown in Ref 27, converts linear motion caused by acceleration into translational motion, which induces two tunnel magnetoresistive sensors to measure similar magnetic field variations caused by two same displacement changes, while the basic mechanical structure of the new device converts the linear motion caused by acceleration into torsional motion, which will induce two tunnel magnetoresistive sensors to measure the differential magnetic field variations caused by two differential displacement changes. The new device helps to suppress the common-mode errors and the interference, which is advantageous to further improve the performance of the device.

**Figure 2:**
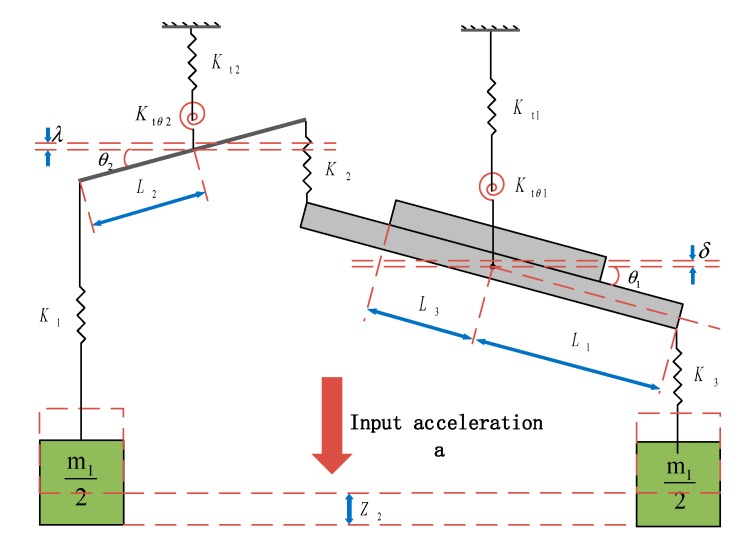
Simplified structural model.

The structural model is appropriately simplified in order to facilitate the theoretical analysis. The simplified structural model of the plane main structure is shown in Figure 2. When the torsional beam stiffness of K_t1_ and K_t2_ along the z-axis is neglected, the equivalent equations are:

(1) $$\frac{m_{1}}{2}a=K_{1}\left(Z_{2}-L_{2}\theta_{2}\right)$$

(2) $$\frac{m_{1}}{2}a=K_{3}\left(Z_{2}-L_{1}\theta_{1}\right)$$

(3) $$K_{t\theta 1}\theta_{1}=L_{1}\left[\frac{m_{1}}{2}a+K_{2}\left(L_{2}\theta_{2}-L_{1}\theta_{1}\right)\right]$$

(4) $$K_{t\theta 2}\theta_{2}=L_{2}\left[\frac{m_{1}}{2}a-K_{2}\left(L_{2}\theta_{2}-L_{1}\theta_{1}\right)\right]$$

where *m*_1_ is the mass of outer proof mass with a unit of kg and *Z*_2_ is the displacement of the outer proof mass with a unit of m. *K*_1_ is the equivalent stiffness of U-suspension beams which connect leverage structures to the outer proof mass; *K*_2_ is the equivalent stiffness of U-suspension beams which connect leverage structures to the inner proof mass; and K_3_ is the equivalent stiffness of linkage structures which connect outer proof mass to the inner proof mass. *K*_1_, *K*_2_ and *K*_3_ have a unit of N/m. *L*_1_ is the equivalent torsion arm length of inner proof mass; *L*_2_ is the torsional arm length of leverage structures; and *L*_3_ is the equivalent torsion arm length of permanent magnetic film. *L*_1_, *L*_2_ and *L*_3_ have a unit of m. *θ*_1_ is the torsional angle of the inner proof mass; *θ*_2_ is the torsional angle of the leverage structures. *θ*_1_ and *θ*_2_ have a unit of rad. *K_tθ_*_1_ is the equivalent torsional stiffness of the torsional beam connected with the inner proof mass, and *K_tθ_*_2_ is the equivalent torsional stiffness of the torsional beam connected with leverage structures. *K_tθ_*_1_ and *K_tθ_*_2_ have a unit of N·m/rad.

The torsional beam stiffness of K_t1_ and K_t2_ has little effect on the final output; therefore, it can be ignored in the derivation of the approximate formula. At the same time, when the solid model is simplified to a lumped parameter model, there will be some approximate errors. However, these approximation errors are small and do not significantly affect the output.

The torsional angle of the inner proof mass *θ*_1_ is

(5) $$\theta_{1}=\frac{m_{1}L_{1}\left[1+\frac{K_{t\theta 2}\left(K_{3}-K_{1}\right)}{2K_{1}K_{3}L_{2}^{2}}\right]}{\frac{L_{1}^{2}}{L_{2}^{2}}K_{t\theta 2}+K_{t\theta 1}}a=\frac{k_{a}}{k}a$$

where

(6) $$k_{a}=m_{1}L_{1}$$

(7) $$k=\frac{\frac{L_{1}^{2}}{L_{2}^{2}}K_{t\theta 2}+K_{t\theta 1}}{1+\frac{K_{t\theta 2}\left(K_{3}-K_{1}\right)}{2K_{1}K_{3}L_{2}^{2}}}$$

where *k_a_* is the equivalent torque coefficient of the input acceleration in the unit of kg·m and *k* is the equivalent torsional elastic stiffness in the unit of N·m/rad. Therefore, the natural frequency of the plane main structure is

(8) $$\omega_{n}=\sqrt{\frac{k}{J}}=\sqrt{\frac{\frac{L_{1}^{2}}{L_{2}^{2}}K_{t\theta 2}+K_{t\theta 1}}{J\left[1+\frac{K_{t\theta 2}\left(K_{3}-K_{1}\right)}{2K_{1}K_{3}L_{2}^{2}}\right]}}$$

where *J* is the rotational inertia of the inner proof mass and the permanent magnetic film in the unit of kg·m^2^, and *ω_n_* is the natural frequency of the plane main structure in the unit of rad/s.

Since the torsion angle of the inner proof mass is minor, the maximum torsional displacement at the diagonal boundary of the permanent magnetic film is

(9) $$Z_{1}=L_{3}\theta_{1}=L_{3}\frac{k_{a}}{k}a$$

The magnetic field distribution along the y-axis due to a rectangular permanent magnetic film can be expressed approximately as *B_y_(x,y,z)* in the unit of T [29]. Only the magnetic field distribution along the y-axis is given, because the sensitive direction of two tunneling magnetoresistive sensors is along the y-axis.

(10) $$B_{y}\left(x,y,z\right)=\frac{\mu_{0}M}{4\pi }\ln\frac{F_{2}\left(-y,x,-z\right)F_{2}\left(y,x,z\right)}{F_{2}\left(y,x,-z\right)F_{2}\left(-y,x,z\right)}$$

where

(11) $$F_{2}(x,y,z)=\frac{\sqrt{{\left(x+a\right)}^{2}+{\left(y-b\right)}^{2}+{\left(z+c\right)}^{2}}+b-y}{\sqrt{{\left(x+a\right)}^{2}+{\left(y+b\right)}^{2}+{\left(z+c\right)}^{2}}-b-y}$$

*M* is the moment density in the unit of T. a, b and c are half of the length along the x-axis, half of the width along the y-axis and half of the thickness along the z-axis of the rectangular permanent magnetic film in the unit of m, respectively. Due to the displacement variation in the z direction, the magnetic field intensity in two diagonal boundaries of the permanent magnetic film can be simplified as

(12) $$B_{yz1}\left(x,y,z\right)=B_{y}\left(x_{0},y_{0},z_{0}\right)+k_{Bz}\Delta z$$

(13) $$B_{yz2}\left(x,y,z\right)=B_{y}\left(x_{0},y_{0},z_{0}\right)-k_{Bz}\Delta z$$

where $k_{Bz}=\frac{\partial B_{y}\left(x,y,z\right)}{\partial z}{|}_{\left(x_{0},y_{0},z_{0}\right)}$ and ∆*z* ≈ *Z*_1_, *k*_Bz_ has a unit of T/m. Two sense displacements are differentially changed, which results in a differential variation in the magnetic field strength. The magnetic field characteristic only gives a rough theoretical derivation. Therefore, a detailed numerical simulation of the magnetic field distribution and the magnetic field change rate is performed using the finite element solid model in subsequent sections.

The output voltages of two tunneling magnetoresistive sensors are

(14) $$V_{1}\approx k_{v}B_{yz1}\left(x,y,z\right)=k_{v}B_{y}\left(x_{0},y_{0},z_{0}\right)+\frac{k_{v}k_{Bz}L_{3}k_{a}}{k}a$$

(15) $$V_{2}\approx k_{v}B_{yz2}\left(x,y,z\right)=k_{v}B_{y}\left(x_{0},y_{0},z_{0}\right)-\frac{k_{v}k_{Bz}L_{3}k_{a}}{k}a$$

where *k_v_* is equivalent to the transforming coefficient of tunneling magnetoresistive sensors from the magnetic field to voltage in the unit of V/T. Then, the output voltage of the interface amplifier circuit is

(16) $$V=k_{amp}\left(V_{1}-V_{2}\right)=\frac{2k_{amp}k_{v}k_{Bz}L_{3}k_{a}}{k}a$$

where *k_amp_* is the equivalent amplification coefficient in the interface amplifier circuit. Obviously, the differential displacement detection method is used significantly to eliminate the influence of the common mode magnetic field in magnetic field detection.

Considering the sensitivity, bandwidth, process realization and other factors of the device, the overall parameter design of the structure is performed. The design methodology is to achieve the maximum sensitivity. In order to accomplish the above purpose, the first aspect is to improve the mechanical displacement sensitivity, which is the mechanical displacement caused by the input acceleration. We can increase the equivalent torque coefficient and reduce the first-order modal frequency. However, the equivalent torque coefficient and the first-order modal frequency must be compatible with the dimension and bandwidth of the device. The second aspect is to improve the sensitivity of the magnetic field, which is the magnetic field variation caused by the displacement change. This is mainly related to the distribution of the magnetic field and the variation rate of the magnetic field caused by the change of the displacement. We can find the maximum magnetic field change rate through simulation and numerical analysis. Finally, the structural design must be compatible with the structural process and overall layout. In fact, we use the subsequent simulation design to optimize the design parameters of the structure to be compatible with the comprehensive design requirements in various aspects. The structure parameters of the tunneling magnetoresistive accelerometer are shown in Table 1.

## 3. Simulation Analysis

**Figure 3:**
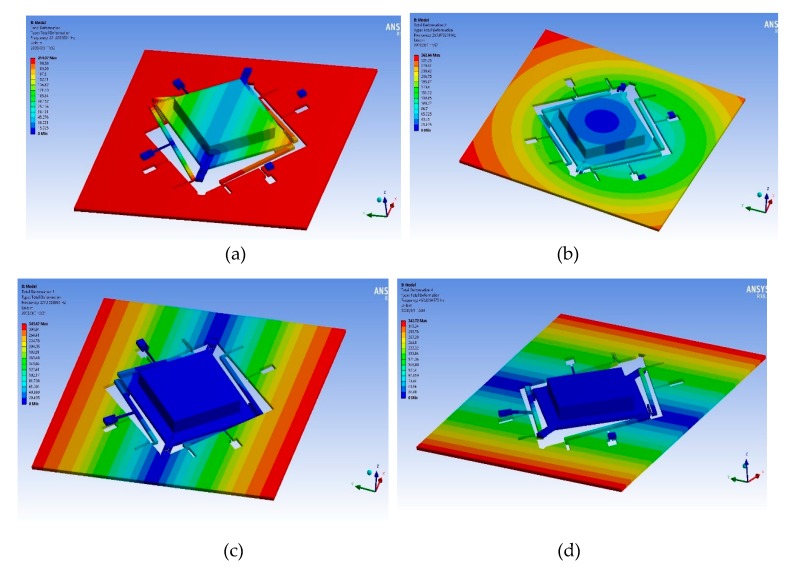
The selected modes of the plane main structure. (**a**) Torsional movement of inner proof mass in first mode. (**b**) Translational movement of inner and outer proof mass along z direction in second mode. (**c**) Torsional movement of outer proof mass in third mode. (**d**) Torsional movement of outer proof mass in fourth mode.

In order to optimize structural performance, the modal simulation of the plane main structure is implemented based on ANSYS software. The torsion of the inner proof mass in the first mode illustrated in Figure 3a is the operating mode of the accelerometer, which has a mode frequency of 221.7 Hz. The outer proof mass moves downward along the z-axis under the inertia force, which simultaneously drives the inner proof mass to twist through leverage structures and linkage structures. The decrease of the first-order mode is beneficial to the improvement of the mechanical sensitivity. The translational movement of the inner and outer proof masses along the z direction in the second mode shown in Figure 3b is the interference mode with a mode frequency of 269.1 Hz. Other interference modes demonstrated in Figure 3c,d are torsional movements of the outer proof mass in the third mode and the fourth mode respectively. The increase of the frequency isolation between the interference modes and the operating mode is beneficial to the interference suppression to the operating mode. Other interference modes are shown in Table 2. 

**Figure 4:**
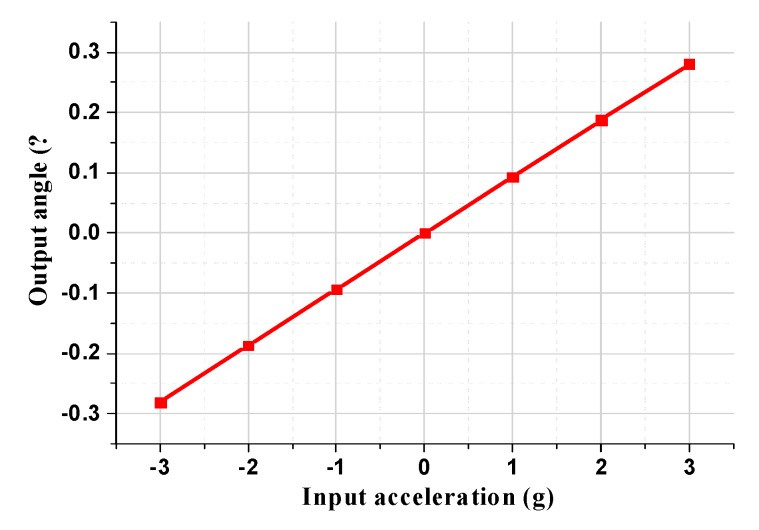
The torsional angle of the permanent magnetic film under different input acceleration.

The torsional angle of the permanent magnetic film under different input accelerations is illustrated in Figure 4. The simulation results demonstrate that the torsional angle of the permanent magnetic film, which has a torsional mechanical sensitivity of 0.094 ^o^ /g, is linearly related to the input acceleration, which indicates that the plane main structure can efficiently convert the input acceleration into the linear torsional displacement of the permanent magnetic film. The maximum displacement mechanical sensitivity at the diagonal boundary of the permanent magnetic film is 3.48 μm/g.

**Figure 5:**
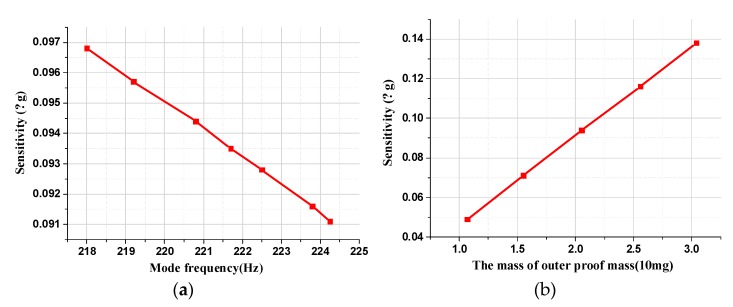
The effect of first-order mode frequency and the outer proof mass on mechanical sensitivity. (**a**) The relationship between mechanical sensitivity and first-order modal frequency. (**b**) The relationship between mechanical sensitivity and the outer proof mass.

The impact of the equivalent torsional elastic stiffness and the equivalent torque coefficient on mechanical sensitivity is analyzed through simulation to optimize structural parameters, shown in Figure 5. 

With the increase in the equivalent torsional elastic stiffness, the first-order mode frequency in Figure 5a improves concomitantly, which results in a monotonous decrease in the mechanical sensitivity. Conversely, the mechanical sensitivity rises with the increase in the outer proof mass, which is positively proportional to the equivalent torque coefficient k_a_, illustrated in Figure 5b. The simulation results are basically consistent with the theoretical formula. Therefore, the decrease in first-order mode frequency and the increase in outer proof mass can effectively improve the mechanical sensitivity in the process of structural optimization. 

**Figure 6:**
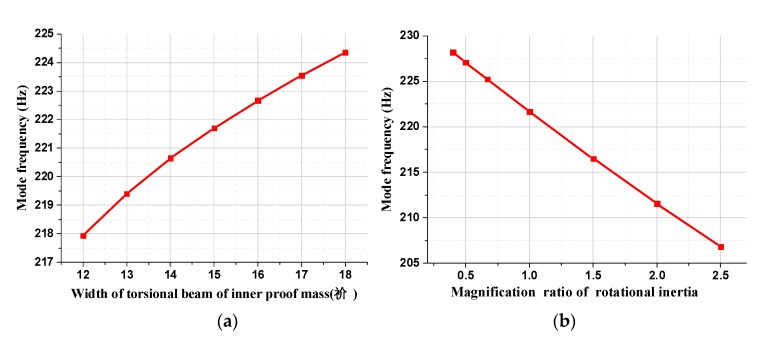
The effect of the width of the torsional beam and the magnification ratio of rotational inertia on the first-order mode frequency. (**a**) The relationship of the first-order mode frequency versus the width of the torsional beam. (**b**) The relationship of the first-order mode frequency versus the magnification ratio of rotational inertia.

The effect of the equivalent torsional elastic stiffness k and the rotational inertia J on the first-order mode frequency is illustrated in Figure 6. With the increase in the width of the torsional beam of the inner proof mass in Figure 6a, the equivalent torsional elastic stiffness k improves consequently due to the rise of the torsional stiffness *K_tθ_*_1_, which leads to the monotonic increase in the first mode frequency. Moreover, the first-order modal frequency decreases with the rise of the amplification ratio of the rotational inertia, as can be seen from Figure 6b. In summary, the above simulation results are in good agreement with the theoretical formula, which confirms the correctness of the theoretical analysis.

**Figure 7:**
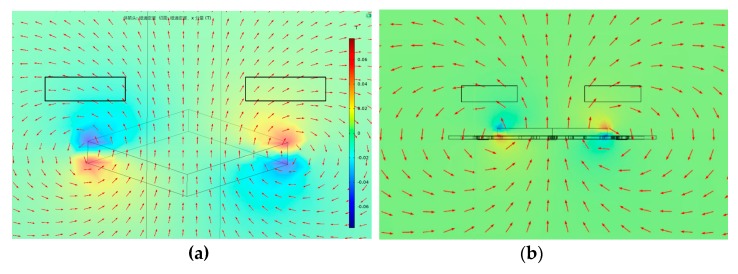
Finite element simulation of magnetic field distribution along y-axis. (**a**) Three-dimensional magnetic field distribution of finite element simulation. (**b**) Magnetic field distribution of finite element simulation in the plane main structure along the y-axis.

To optimize the layout of the tunneling magnetoresistive sensors for maximum magnetic field sensitivity, the magnetic field characteristic is analyzed by the finite element simulation based on the solid model. The finite element model of the physical structure, which is constructed by the Comsol software according to the parameters shown in Table 1, is used to solve the magnetic field distribution of the tunneling magnetoresistive sensors, illustrated in Figure 7. The dimension of the cuboid permanent magnetic film is set to 3000 μm (length) × 3000 μm (width) × 500 μm (height). Theoretically, the outer space of the cuboid permanent magnet film is an infinite area. However, a spherical air model with a radius of 150 mm is constructed around the cuboid permanent magnet film for the research simplification. The relative permeability of the sphere air model is set to 1. We define the magnetic field environment with a temperature of 293.15 K, an absolute pressure of 1atm and the permanent magnet film magnetization of 198,944 A/m. Since the sensitive axis of the tunneling magnetoresistive sensor is on the y-axis, only the magnetic field characteristic along the y-axis direction is extracted. The magnetic field simulation results demonstrate that the magnetic field above the center of the permanent magnetic film is practically vertical with the y-axis. Therefore, the component of the magnetic field is almost zero in the horizontal direction. However, the magnetic field at the diagonal boundary of the permanent magnetic film is almost horizontal, which indicates that the maximum horizontal magnetic field intensity can be detected at this location.

**Figure 8:**
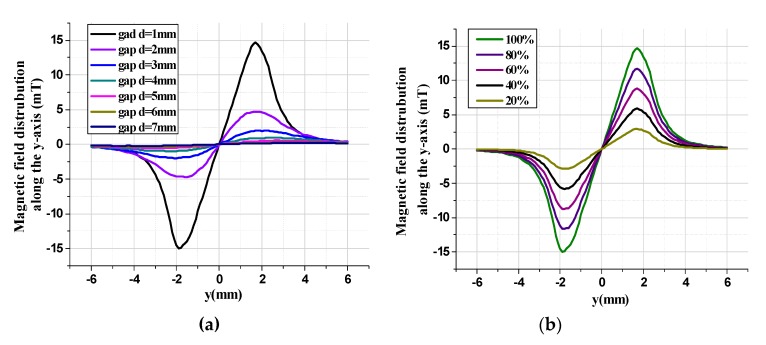
Magnetic field characteristic along y-axis under various conditions. (**a**) Magnetic field distribution versus different vertical gaps. (**b**) Magnetic field distribution versus different magnetic field strength (d = 1 mm).

The magnetic field intensity characteristic along the y-axis is simulated under various conditions. Figure 8a shows the magnetic field distribution along the y-axis under different vertical gaps in the tunneling magnetoresistive sensor. The simulation results illustrate that the magnetic field intensity along the y-axis reaches the maximum at the diagonal boundary of the permanent magnetic film and almost zero at the center of the permanent magnetic film. Moreover, the magnetic field strength has central symmetry. In addition, the maximum magnetic field intensity decreases from 14.93 mT to 0.22 mT as the vertical gaps between the tunneling magnetoresistive sensor and the permanent magnetic film increase from 1 mm to 7 mm. The magnetic field distribution along the y-axis in the tunnel magnetoresistive sensor is related to the magnetic properties of the permanent magnetic film, as reflected in Figure 8b. When the magnetic field strength of the permanent magnetic film reduces from 100% to 20%, the maximum magnetic field intensity in the tunneling magnetoresistive sensor diminishes from 14.93 mT to 2.89 mT, which indicates that the magnetic field intensity in the tunneling magnetoresistive sensor is positively correlated with the magnetic field strength of permanent magnetic film.

**Figure 9:**
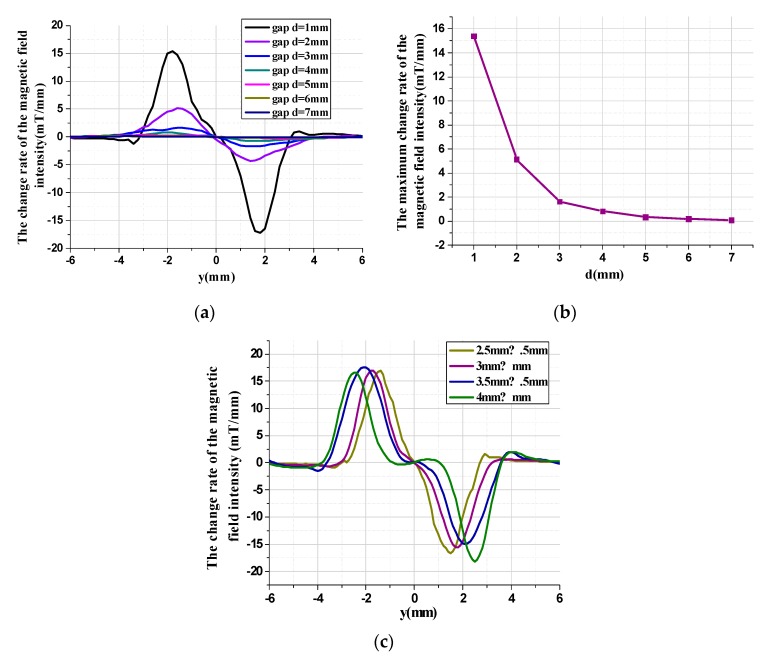
The change rate of the magnetic field intensity along y-axis due to a displacement variation in the z direction in various conditions. (**a**) The change rate of the magnetic field intensity under different vertical gaps. (**b**) The maximum change rate of the magnetic field intensity under different vertical gaps. (**c**) The change rate of the magnetic field intensity under different structure dimensions of permanent magnet film.

The variation in the structure dimension of the permanent magnetic film and the vertical gap between the tunneling magnetoresistive sensor and the permanent magnetic film affect not only the distribution of the magnetic field intensity but also the distribution of the change rate of the magnetic field intensity, shown in Figure 9. 

Figure 9a illustrates the change rate of the magnetic field intensity in the y-axis due to the displacement variation in the z direction under different gaps. Similarly, the change rate of the magnetic field intensity is of central symmetry in the permanent magnetic film. The change rate of the magnetic field intensity is almost zero at the center of the permanent magnetic film, which indicates that the tunneling magnetoresistive accelerometer has the minimum sensitivity in that position. However, the maximum change rate of the magnetic field intensity in y-axis due to the displacement variation along the z direction can be obtained at the boundary of the permanent magnetic film. Therefore, the optimal location with the maximum magnetic field sensitivity for the tunneling magnetoresistive sensors is directly above the diagonal boundary of the permanent magnetic film. Two diagonal boundaries of the permanent magnetic film twist in opposite directions under the inertial acceleration and have opposite displacement sensitivities. Two tunneling magnetoresistive sensors are arranged directly above two diagonal boundaries to realize differential detection for the magnetic field intensity variation due to the displacement variation along the z direction. The maximum change rate of the magnetic field in the tunneling magnetoresistive sensor, illustrated in Figure 9b, decreases from 15.4 mT/mm to 0.09 mT/mm when the vertical gaps increase from 1 mm to 7 mm. The structure dimension of the permanent magnetic film has a significant effect on the distribution characteristic of the magnetic field, shown in Figure 9c. Obviously, the location of maximum magnetic field intensity change rate, which lies around the diagonal boundary of the permanent magnetic film, shifts with the dimension amplification of the permanent magnetic film.

The deviation of the numerical simulation with a fairly good accuracy is only related to the truncation error of the software. The only factor that cannot be accurately estimated and simulated comes from the measurement of the tunneling magnetoresistive sensor. The tunnel magnetoresistive sensor does not measure the magnetic field at a single point, but at a local area. Therefore, the final characteristic can only be obtained through experimental results.

## 4. Measurement and Control Circuit

**Figure 10:**
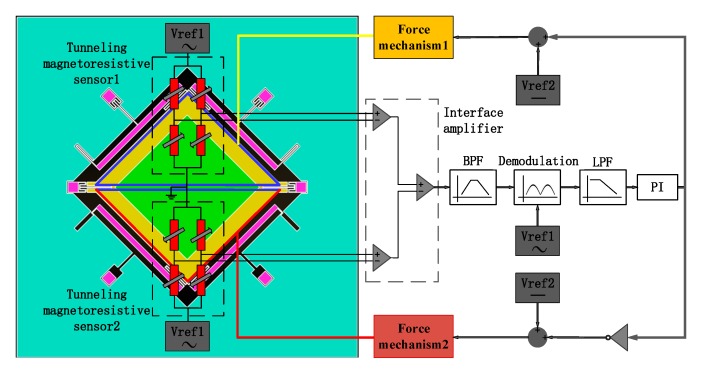
The scheme of the measurement and control circuit.

The scheme of the measurement and control circuit is shown in Figure 10. The core structure located in the center of the tunneling magnetoresistive sensor is a Wheastone bridge composed of four symmetrical tunneling magnetic resistances. The tunneling magnetic resistances arranged diagonally have the same sensitive direction of magnetic field, while the tunneling magnetic resistances arranged adjacently have opposite sensitive directions of the magnetic field. The tunnel magnetoresistive sensors are driven by AC voltage with 1 kHz frequency superimposed with a DC voltage reference, which modulates the detection voltage signal to 1 kHz frequency, thereby suppressing the interference of the low-frequency noise. The steady-state output voltage of tunneling magnetoresistance Sensor 1 is equal to that of tunneling magnetoresistance Sensor 2 without the acceleration input, which will produce no significant signal output from the interface amplifier. When the acceleration is inputted, the output voltage variation of the tunneling magnetoresistance Sensor 1 is opposite to that of tunnel magnetoresistance Sensor 2, which will result in an output signal for the subsequent circuits processing, including a band pass filter (BPF), the demodulator circuit, a low pass filter (LPF) and a proportional-integral (PI) controller. The output voltage of PI, superimposed with a DC voltage reference (Vref2), is connected to the feedback electrodes through two electrostatic force mechanisms, so as to pull the inner proof mass back to the equilibrium state and realize the closed-loop detection of acceleration. 

The closed-loop feedback principle is similar to a conventional capacitive accelerometer. The difference is that the capacitive accelerometer measures the displacement variation of the proof mass through the capacitance, while the tunneling magnetoresistive accelerometer uses two differential tunnel magnetoresistive sensors to detect the torsional displacement of the inner proof mass. The circuit then drives the integrator output feedback voltage through subsequent demodulation and filtering modules. The increase in the input acceleration will enlarge the output voltage of integrator. Similar to the capacitive accelerometer, the tunneling magnetoresistive accelerometer uses a capacitor between the inner proof mass and the bottom feedback electrodes to form an electrostatic capacitive torque device. A reverse torsional restoring force of the inner proof mass, which is opposite to the input acceleration motion, can be generated to implement the feedback force by applying a feedback voltage on the capacitor, which ultimately drives the outer proof mass back to the equilibrium position by the inner proof mass.

## 5. Experiment

**Figure 11:**
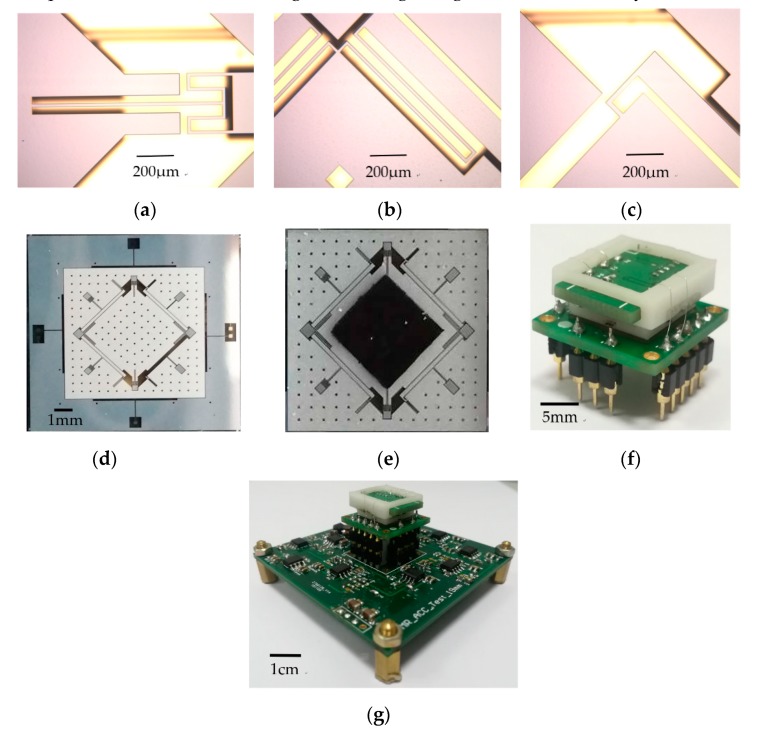
The optical micrograph of the fabricated tunnel magnetoresistive accelerometer. (**a**) The torsional beam. (**b**) U-suspension beam. (**c**) L-suspension beam. (**d**) The plane main structure. **(e)** The plane main chip bonded with the permanent magnetic film. (**f**) Microassembly structure with the tunneling magnetoresistive sensors. (**g**) The prototype of the tunneling magnetoresistive accelerometer.

The standard deep dry silicon on glass (DDSOG) process was utilized to fabricate the plane main structure, to verify the principle of the micromachined z-axis tunneling magnetoresistive accelerometer. The prototype of the tunneling magnetoresistive accelerometer was implemented through micro-assembly of the plane main structure, the permanent magnetic film and tunneling magnetoresistive sensors, as illustrated in Figure 11. The fabrication and micro-assembly processes are shown in Table 3.

The bonding anchors were firstly patterned and exposed by lithography in a monocrystalline wafer with 200-μm thickness, then were etched by the deep reactive ion etching (DRIE) to form the steps of bonding anchors with 10-μm height. A Cr/Ti/Au stack layer was sputtered in a Pyrex glass substrate with 500-μm thickness to manufacture the electrode wires and pads. An electrostatic anodic bonding process was utilized to combine the silicon wafer with the Pyrex glass wafer, then the silicon wafer was thinned to 120 μm thickness by a wet etching process with KOH solution. Subsequently, a lithography and DRIE process was used to pattern and etch to release the silicon wafer. The fabricated plane main structure had dimensions of 8000 μm (length) × 8000 μm (width) × 120μm (height), as shown in Figure 11d. Some structural fabrication details are demonstrated in Figure 11a–c. A permanent magnetic film of 3000 μm (length) × 3000 μm (width) × 500 μm (height) with a moment density of 250 mT was bonded on the plane main chip structure by silica gel and accurately aligned with the micro-markers based on the microscope platform, as shown in Figure 11e. The damping holes on the plane main structure were used to accurately calibrate the position of the permanent magnetic film for accurate positioning.

A frame based on 3D printing was combined adhesively to the plane main chip structure. Finally, two tunneling magnetoresistance sensors in opposite detecting directions welded on the printed circuit board (PCB) board were micro-assembled in the frame, shown in Figure 11f. Additionally, two tunneling magnetoresistive sensors were arranged directly above the diagonal boundary of the permanent magnetic film with the center aligned accurately. We adopted two commercial linear tunneling magnetoresistive sensors of TMR9001 in Multi-Dimension Technology, with a sensitivity of 300 mV/V/Oe and a noise floor of 150 pT/Hz^0.5^, to measure the magnetic field variation caused by acceleration input [30]. Moreover, the height of the 3D printing frame is accurately adjusted to guarantee that the tunneling magnetoresistive sensors operate in the unsaturated region with high magnetic field sensitivity.

**Figure 12:**
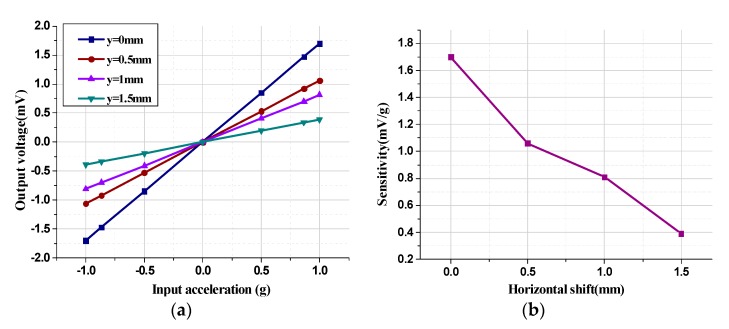
Acceleration input and output response characteristics of the tunneling magnetoresistive accelerometer under various horizontal shifts. (**a**) Output voltage versus input acceleration under various horizontal shifts. (**b**) Sensitivity versus various horizontal shifts.

The system experiments under various conditions were implemented to evaluate the performance of the micromachined z-axis tunneling magnetoresistive accelerometer. Since the relative shift between the tunnel magnetoresistance sensors and the permanent magnetic film along the y-axis has a great influence on the sensitivity, the acceleration input and output response characteristics under various horizontal shift along the y-axis were implemented as illustrated in Figure 12. The vertical gap between the tunnel magnetoresistance sensors and the permanent magnetic film was set to 1 mm and the permanent magnetic film had a thickness of 0.5 mm. Obviously, the horizontal shift between the diagonal boundary of the permanent magnet film and the center of the tunnel magnetoresistive sensors was negatively correlated with the sensitivity of the tunneling magnetoresistive accelerometer. With the increase in the horizontal shift y from 0 mm to 1.5 mm, the sensitivity decreased from 1.7 mV/g to 0.3 9mV/g, which verifies that the horizontal shift of y=0mm is the optimal horizontal layout for the tunnel magnetoresistive sensors. 

**Figure 13:**
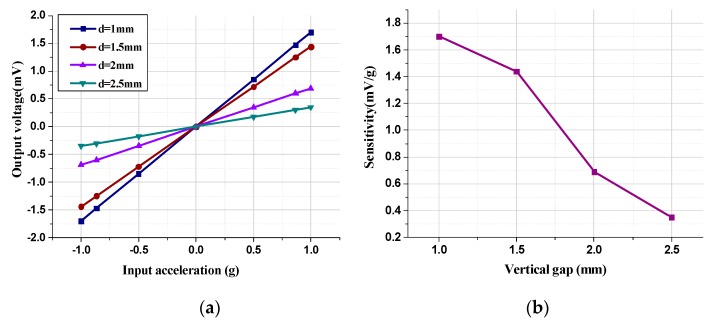
Acceleration input and output response characteristics of the tunneling magnetoresistive accelerometer under different vertical gaps. (**a**) Output voltage versus input acceleration under different vertical gaps. (**b**) Sensitivity versus different vertical gaps.

Similarly, the acceleration input and output response characteristics under different vertical gaps were implemented with a horizontal shift of 0 mm and a permanent magnetic film thickness of 0.5 mm, as described in Figure 13.

The sensitivity diminishes from 1.7 mV/g to 0.35 mV/g as the vertical gap increases from 1 mm to 2.5 mm, which demonstrates that the decrease in the vertical gap can effectively improve the sensitivity. However, the decrease in the vertical gap will cause an increase in the magnetic field intensity around the tunneling magnetoresistance sensors. The vertical gap must be controlled within a certain range in order to avoid the saturation zone of the magnetic field and the failure of the detection function in the tunneling magnetoresistance sensors. Ultimately, the system experiment results demonstrate that a vertical gap d = 1 mm is a feasible arrangement for the tunneling magnetoresistance sensors to operate linearly in the unsaturated magnetic field region with high sensitivity.

**Figure 14:**
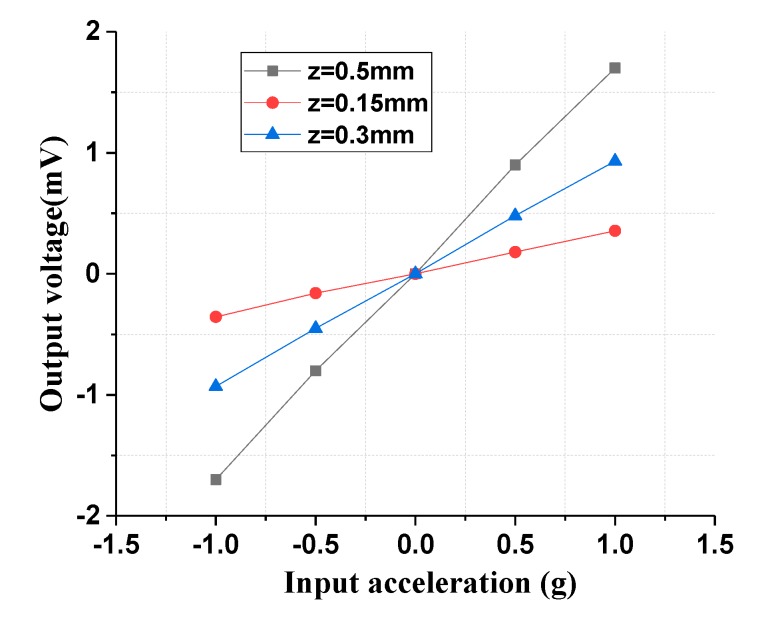
Acceleration input and output response characteristics under various thicknesses of the permanent magnet film.

The thickness variation of the permanent magnetic film also has significant influence on the sensitivity of the tunneling magnetoresistance accelerometer. Therefore, the acceleration input and output response characteristic in different thicknesses of the permanent magnetic film was implemented with a vertical gap of 1 mm and a horizontal shift of 0 mm. As the thickness of the permanent magnetic film increased from 0.15 mm to 0.5 mm, the sensitivity of the tunneling magnetoresistive accelerometer was improved from 0.36 mV/g to 1.7 mV/g, as shown in Figure 14. An increase in the thickness of the magnetic film will lead to a rise in the magnetic field intensity, which will increase the sensitivity of the tunneling magnetoresistive sensors even in the same detection position. Simultaneously, the increase in thickness will result in an increase in the mass of the permanent magnetic film, which will further increase the sensitivity of the mechanical displacement. 

Sensitivity is affected by three aspects. One is the change sensitivity of the mechanical displacement caused by the input acceleration. The second is the sensitivity of the magnetic field variation caused by the displacement change, and the third aspect is the magnetic field sensitivity of the tunneling magnetoresistive sensor. Mechanical sensitivity can be improved by modifying the dimension of the elastic beam, but too-high mechanical sensitivity will reduce the measurement range, and it will be susceptible to external shock and vibration. The magnetic field sensitivity of the tunneling magnetoresistive sensor is related to the material and structure of the tunnel magnetoresistive layer, which is difficult to further improve. Therefore, the sensitivity of the magnetic field variation caused by the displacement change has a large impact on the sensitivity of the entire device. Although we know that the maximum sensitivity of the magnetic field variation caused by the displacement change is at the magnetic field boundary, the tunneling magnetoresistive sensor does not measure the magnetic field at a single point, but over a local area. Therefore, the area of the diagonal magnetic sheet boundary is too small. At the same time, due to the torsional movement of the proof mass, the displacement of the diagonal magnetic sheet boundary linearly decays. Both factors reduce the sensitivity of the magnetic field variation caused by the displacement change.

**Figure 15:**
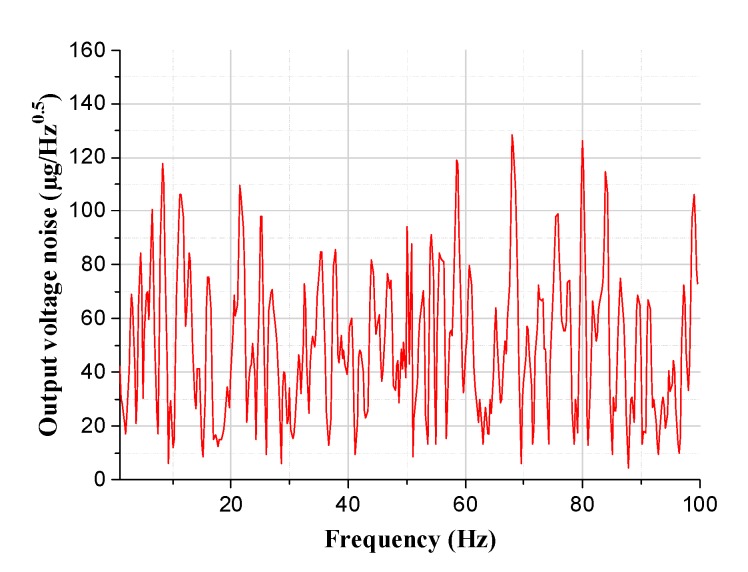
Output voltage noise spectrum of the z-axis tunneling magnetoresistive accelerometer.

Finally, acceleration resolution measurement of the z-axis tunneling magnetoresistive accelerometer was implemented to evaluate the performance of the prototype. The output voltage noise spectrum demonstrates that the acceleration resolution of the prototype is about 128 μg/Hz^0.5^, as shown in Figure 15. Simultaneously, the performance comparisons of tunneling magnetoresistive accelerometers recently reported are shown in Table 4. This work has certain advantages in the miniaturization and integration of the device but needs further improvement in device sensitivity and noise performance. 

In conclusion, the above comprehensive experiments demonstrate that the scheme of the z-axis tunneling magnetoresistive accelerometer is feasible and achieves a considerable performance.

## 6. Conclusions

This paper described the design, simulation, fabrication and testing of a novel micromachined z-axis torsional accelerometer based on the tunneling magnetoresistive effect, which is mainly composed of the top substrate, the middle plane main structure and the bottom substrate. The middle plane main structure, which is driven to twist for pushing the deflection of the permanent magnet film, is used to transform the input acceleration into a variation in magnetic field intensity. Two differential tunneling magnetoresistive sensors, bonded on the top substrate directly above two diagonal boundaries of the permanent magnetic film, are adopted to measure the magnetic field variation. The plane main structure is reverted to the equilibrium state and realizes the closed-loop detection of acceleration by the electrostatic feedback electrodes plated on the bottom substrate. We constructed the finite element model of the plane main structure and optimized the structural mode and the mechanical sensitivity to verify theoretical formula correctness based on ANSYS software. Simultaneously, the layout of the tunneling magnetoresistance accelerometer was analyzed to optimize the magnetic field characteristic by the finite element simulation based on Comsol software. The plane main chip structure was fabricated by the standard deep dry silicon on glass (DDSOG) process, and had a dimension of 8000 μm (length) × 8000 μm (width) × 120 μm (height). A prototype of the micromachined z-axis tunneling magnetoresistive accelerometer was implemented by the micro-assembly of the plane main structure, the permanent magnet film and two tunneling magnetoresistive sensors. The experiment results demonstrate that the prototype has a maximal sensitivity of 1.7 mV/g and an acceleration resolution of 128 μg/Hz^0.5^ in z-axis sensitive direction.

## Figures and Tables

**Table 1:** Structure parameters.

Parameter	Value	Parameter	Value
Outer proof mass (kg)	1.02 × 10^−5^	U-suspension beam (length × width (μm))	851 × 15
Mode frequency ω_n_ (rad/s)	1392.98	L-suspension beam (length × width (μm))	203 × 15
Length 2a (μm)	3000	Torsional beam (length × width (μm))	972 × 15
Width 2b (μm)	3000	Leverage(length × width (μm))	3466 × 150
Thickness 2c (μm)	500	Gap d1(between proof mass and feedback electrode (μm) )	10
Inner proof mass (length × width(μm))	4000 × 4000	Feedback electrode area (mm^2^)	12.16
Outer proof mass (length × width(mm))	8000 × 8000	Gap d2(between tunnel magnetoresistive sensor and proof mass (μm) )	1000
Thickness of main structure(μm)	120	Moment density M(mT)	250

**Table 2:** The first six mode frequencies of the plane main structure.

Modal	1	2	3	4	5	6
Frequency (Hz)	221.7	269.1	279.1	468	510.2	794.3

**Table 3:** The fabrication and micro-assembly processes.

(a) Coating the photoresist	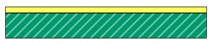
(b) Etching structural anchors on the silicon wafer	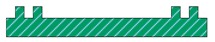
(c) Etching the glass groove	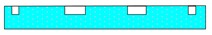
(d) Sputtering the Cr/Ti/Au electrodes	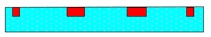
(e) Silicon–glass anode bonding	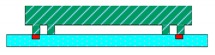
(f) Thinning the silicon structure layer	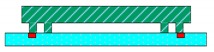
(g) DRIE etching to release structure	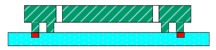
(h) Pasting the tunnel magnetoresistive sensor	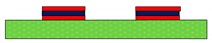
(i) Micro-assembling the permanent magnet film by silica gel	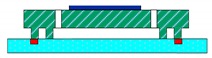
(j) Micro-assembling the tunnel magnetoresistive sensor with silicon structure using frame	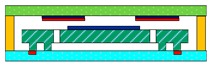

**Table 4:** Performance comparison of tunneling magnetoresistive accelerometers recently reported.

Parameter	Ref [25] Phan (2008)	Ref [26] Yang (2019)	Ref [27] Yang (2019)	Ref [28] Yao (2019)	This Work
**Structural integration**	High	Low	Medium	Low	Medium
**Core structure area (mm^2^)**	(1.5 × 1.5) × 3.14/4 (1.5mm diameter)	17 × 17	6.4 × 6.4	90 × 50	8 × 8
**Sensitivity** **(mV/g)**	0.96	401.3	8.85	1145	1.7
**Noise floor** **(ug/Hz^0.5^)**	35	20	86.2	NA	128
